# Functional analysis of *Candida albicans* GPI-anchored proteins: Roles in cell wall integrity and caspofungin sensitivity

**DOI:** 10.1016/j.fgb.2008.08.003

**Published:** 2008-10

**Authors:** Armêl Plaine, Louise Walker, Gregory Da Costa, Héctor M. Mora-Montes, Alastair McKinnon, Neil A.R. Gow, Claude Gaillardin, Carol A. Munro, Mathias L. Richard

**Affiliations:** a Laboratoire de Microbiologie et Génétique Moléculaire, AgroParisTech, UMR-INRA 1238, UMR-CNRS 2585, 78850 Thiverval-Grignon, France; b School of Medical Sciences, Institute of Medical Sciences, University of Aberdeen, Foresterhill, Aberdeen AB25 2ZD, UK

**Keywords:** *Candida albicans*, GPI, Calcofluor, Cell wall, Surface

## Abstract

The outer layer of the *Candida albicans* cell wall is enriched in highly glycosylated proteins. The major class, the GlycosylPhosphatidylInositol (GPI)-anchored proteins are tethered to the wall by GPI-anchor remnants and include adhesins, glycosyltransferases, yapsins and superoxide dismutases. *In silico* analysis suggested that *C. albicans* possesses 115 putative GPI anchored proteins (GpiPs), almost twice the number reported for *Saccharomyces cerevisiae*. A global approach to characterise *in silico* predicted GpiPs has been initiated by generating a library of 45 mutants. This library was subjected to a screen for cell wall modifications by testing the cell wall integrity (SDS and Calcofluor White sensitivity) and response to caspofungin. We showed that, when caspofungin sensitivity was modified, in more than half of the cases the susceptibility can be correlated to the level of chitin and cell wall thickness: sensitive strains have low level of chitin and a thin cell wall. We also identified, for the first time, genes that when deleted lead to decreased caspofungin sensitivity: *DFG5, PHR1, PGA4* and *PGA62*. The role of two unknown GpiPs, Pga31 and Pga62 in the cell wall structure and composition was clearly demonstrated during this study.

## 1 Introduction

*Candida albicans* is the most common fungal pathogen of humans. A crucial feature of this microorganism is its ability to survive in the host and to infect several anatomically distinct sites. This fungus has elaborated numerous mechanisms to adapt to a diversity of niches (mouth, digestive tract, skin, vagina, etc.) and resides in a healthy host without causing overt disease. As an opportunistic pathogen, *C. albicans* can invade immunocompromised hosts and cause life threatening diseases that may arise as hematogenously disseminated infections or as localized primary diseases of deep organs (Calderone and Fonzi, 2001). Each of these niches imposes diverse and differing stresses on the fungus, including variable pH and temperature, nutrient limitation, and immune system defences. Accordingly, the cell has to adapt constantly to cope with these varied and changing environmental conditions. In fungi, the cell wall is essential for maintaining the osmotic balance of the cell, for creating and maintaining its shape during morphogenesis and providing it with protection from environmental stresses (Klis et al., 2002). In addition, in pathogenic fungi, the cell wall and especially cell wall proteins are known to play a key role in the relationship between the fungal cell and the host, contributing to host tissue adhesion and to immune response modulation (Ruiz-Herrera et al., 2006).

The *C. albicans* cell wall is a network composed primarily of β-1,3- and β-1,6-glucans, chitin and mannoproteins. The major class of cell wall proteins has the sequence features of GlycosylPhosphatidylInositol (GPI)-anchored proteins, including an N-terminal signal sequence and a C-terminus containing a GPI anchor attachment site (ω-site). GPI anchored proteins (GpiPs) are linked to the plasma membrane via a preformed GPI anchor that is added to the protein in the endoplasmic reticulum by a transamidase enzyme complex (Tiede et al., 1999). In some fungi, several results suggest that a subset of GpiPs are cleaved from the membrane and translocated to the cell wall where they are linked covalently to β-1,6-glucan (Hamada et al., 1999). By comparing *in silico* predicted GpiPs reported in a number of studies (de Groot et al., 2003; Eisenhaber et al., 2004; Garcera et al., 2003), we estimate 115 putative GpiPs in the *C. albicans* genome (Richard and Plaine, 2007). Almost twice as many putative GpiPs as in *S. cerevisiae* and more than 65% of these predicted GpiPs are of unknown function (Richard and Plaine, 2007). Throughout this article the proteins are described as GPI anchored having considered the consistent published *in silico* predictions (de Groot et al., 2003; Eisenhaber et al., 2004; Garcera et al., 2003; Richard and Plaine, 2007). Nevertheless, a biochemical analysis of each protein would be necessary to confirm they are indeed modified by the addition of a GPI-anchor.

Although the majority of the research on fungal GPI-anchoring had been performed in *S. cerevisiae*, several biochemical studies have provided evidence of GPI-anchoring in *C. albicans* (Grimme et al., 2004; Kapteyn et al., 2000; Kapteyn et al., 1994). Kapteyn and collaborators have described the cross-linkage of GPI proteins to cell wall β-1,6-glucan in *C. albicans* (Kapteyn et al., 1994). Grimme et al. (2004) demonstrated that the *C. albicans* mannosyltransferase Smp3, involved in the synthesis of the GPI-anchor glycan, retained the same function as its *S. cerevisiae* ortholog. In addition, the inability to generate a *CaSMP3* null mutant suggested the synthesis of GPI-anchors is essential in *C. albicans* as it is in *S. cerevisiae* (Grimme et al., 2004).

A previous study in our laboratory established that GpiPs were no longer normally linked to the cell wall in a *C. albicans gpi7*−/− mutant affected in GPI-anchor side chain addition. This mis-localization of numerous cell wall proteins had dramatic consequences on cell wall composition and the *in vivo* virulence of the mutant (Richard et al., 2002a; Richard et al., 2002b). These data highlight the importance of GpiPs in the interaction with the host, but does not differentiate a direct role from an indirect role. A better understanding of the functions associated with this class of protein might thus lead to the identification of potential targets for therapeutic treatment of *C. albicans*.

To date, several strategies have permitted the identification of GpiPs in *C. albicans*, including proteomics, functional expression in *S. cerevisiae*, cloning of surface antigens, and identification of *C. albicans* sequence homologs of characterized *S. cerevisiae* GpiPs (de Groot et al., 2004; Garcera et al., 2003; Lamarre et al., 2000). Here, we report the first systematic functional analysis of a large number of putative GpiPs in the human pathogen *C. albicans*. A total of 45 GpiP mutants were constructed or gathered from different laboratories and submitted to a series of phenotypic tests in order to characterize their involvement in cell wall structure. Our results extend the functional analyses of putative GpiPs characterized elsewhere and identify several novel putative GpiPs that contribute to cell wall integrity.

## 2 Methods

### 2.1 Strains

*Escherichia coli* DH5α strain was used for plasmid propagation. The mutant strains used in this study are presented Table 1. All mutants were Ura^+^, Arg^+^ and His^−^ were derived from strain BWP17 (*ura3::imm434/ura3::imm434, his1::hisG/his1::hisG, arg4::hisG /arg4::hisG*) (Wilson et al., 1999). In all experiments, two independent mutant clones were tested twice and compared to DAY286 (*ura3::imm434/ura3::imm434, his1::hisG/his1::hisG, pARG4::URA3::arg4::hisG/arg4::hisG*), a Ura^+^, Arg^+^, His^−^ derivative of BWP17 (Davis et al., 2000), unless otherwise indicated. Mutants obtained by the classical URA-blaster method (Fonzi and Irwin, 1993) and lacking either *CSA1, HWP1, HYR1, PHR1, PHR2, RBT1, RBT5, SAP10, SOD5* and *SSR1* along with the corresponding complemented or reference strains, were obtained from the groups who generated them (see Table 1). For these latter strains, all experiments were performed two to four times and SC5314 or CAI-4 (Fonzi and Irwin, 1993) was used as reference strains according to their genetic background.

### 2.2 Culture media

DH5α was grown at 37 °C in Luria–Bertani medium supplemented with 100 μg ampicillin mL^−1^ (Amp) and 20 μg kanamycin mL^−1^ (Kan) as required. *C. albicans* strains were grown in YPD plus uridine (2% dextrose, 2% Bacto peptone, 1% yeast extract and 80 μg uridine mL^−1^) at 30 °C. Following transformation, clones were selected on synthetic medium (SC; 2% glucose, 0.67% yeast nitrogen base without amino acid plus ammonium sulphate, 1.7 g amino acid drop out mix L^−1^, supplemented for auxotrophic requirements).

### 2.3 Mutant library construction

**Fig. 1:**
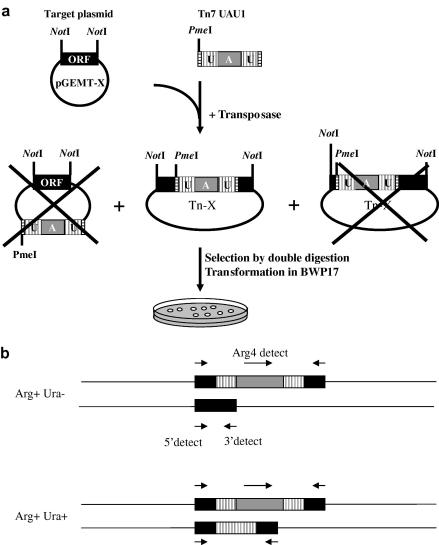
Strategy used for gene interruptions (the ORF to be disrupted is depicted as a black box, the *ARG4* marker as a grey box, the *URA3* marker as a vertically hatched box and the IR of the Tn7 transposon as horizontally hatched boxes). (a) Construction of the disruption cassette by *in vitro* transposition. The integration site of the *Tn7-UAU1* cassette was estimated by double digestion (PmeI and NotI). The target plasmid which had integrated the Tn7-UAU1 transposon in the middle of the ORF was selected and transformed in the *C. albicans* strain BWP17. (b) Selection of double disruptions by colony PCR. Colonies isolated on SC-Arg-Ura plates were screened for the absence of the wild-type allele and the presence of the UAU1 insertion allele. Primers 5′ and 3′ detect were the primers used to amplify the ORF in order to create the target plasmid.

All *C. albicans* sequences were obtained from the Candida DataBase and the Candida Genome Database web sites (http://genolist.pasteur.fr/CandidaDB/ and http://www.candidagenome.org/). The open reading frame (ORF) of all genes to be disrupted was amplified by PCR and ligated into the vector pGEM-T (Promega, Madison, WI, USA) to generate the target plasmid for subsequent *in vitro* transposition using the GPS-M mutagenesis kit (New England Biolabs, Beverly, MA, USA, see Fig. 1). The donor plasmid, pAED98 contains *Tn7*-UAU1 (Davis et al., 2002), a modified *Tn7* transposon carrying the UAU1 cassette (Enloe et al., 2000). *In vitro* transposition was performed using 20 ng of pAED98 and 80 ng of the target plasmid. Transposon-mutagenized plasmids were transformed into DH5α and transformants were selected on LB + Amp + Kan plates. For each transposition, 24 plasmids were isolated from individual colonies using the Millipore Montage miniprep system (Millipore, Bedford, USA). In order to map the integration site of the *Tn*7-UAU1 in the target plasmid, we digested the mutagenized plasmid with NotI and PmeI. One target plasmid, which had integrated the *Tn7*-UAU1 transposon near the middle of the ORF was selected for each gene (see Fig. 1a).

### 2.4 Construction of homozygous mutants

Interruption of both alleles of each *C. albicans* gene was achieved following the UAU1 method (Enloe et al., 2000). Briefly, the *Tn7*-UAU1 mutagenized plasmid was digested with NotI to release the UAU1 disruption cassette which was then used to transform *C. albicans* strain BWP17 (Wilson et al., 1999). Arg^+^ transformants were selected. Genotypes were analysed by colony PCR to detect the wild-type allele and the *Tn7*-UAU1 insertion (see Fig. 1b). For each plasmid, 24 heterozygous strains were patched onto YPD plates, grown at 30 °C for 2 days. After replication onto SC-Arg-Uri plates, the colonies were screened by colony PCR for the absence of the wild-type allele and the presence of the UAU1 insertion allele. Genotypes of all transformants showing a phenotype different from the control strain were further confirmed by Southern analysis.

### 2.5 Phenotypic analysis by drop tests

The phenotypes of the 45 GpiP mutants were monitored using drop tests. All media were supplemented with histidine. Strains were grown overnight in YPD at 30 °C and diluted with H_2_O to an OD_600_ of 1. Then, 5 μl of serial 1/10 dilutions was spotted onto the following solid media: YPD supplemented with 0.025% SDS or 30 μg Calcofluor White mL^−1^ (Sigma–Aldrich, Steinheim, Germany). Plates were monitored over 2 days for altered growth.

### 2.6 Antifungal tests

The *in vitro* susceptibility of the mutants was tested with caspofungin. The Minimal Inhibitory Concentration (MIC) was determined using the microbroth dilution method M27-A2 (NCCLS, 2002). Each drug was obtained from its respective manufacturer as pure compound. RPMI medium (Sigma–Aldrich, Steinheim, Germany) buffered at pH 7 with morpholinepropanesulfonic acid (MOPS; Sigma–Aldrich, Steinheim, Germany) was used. Additional tests with caspofungin were performed using the drop test method on SC plates buffered at pH 7.0 with HEPES 150 mM and containing sub-inhibitory concentrations of caspofungin. Plates were incubated for two days at 37 °C.

### 2.7 Chitin content quantification

Cell walls were prepared from exponential *C. albicans* yeast cultures grown in YPD and the chitin content was measured as described previously with modifications (Munro et al., 2003). Briefly, cell walls were extracted by disrupting cells with glass beads (Sigma, G9268) using a Fastprep cell breakage machine (Thermo Savant, Middlesex, UK). Samples were then washed five times with 1 M NaCl and cell walls were extracted using SDS-MerOH buffer (50 mM Tris, 2% sodium dodecyl sulphate (SDS), 0.3 M β-mercaptoethanol, 1 mM EDTA; pH 8.0) at 100 °C for 10 min. Cell wall pellets were resuspended in sterile *d*H_2_O, freeze dried, and the dry weight of recovered cell walls was measured. Chitin contents were determined by measuring the glucosamine released by acid hydrolysis of purified cell walls (Kapteyn et al., 2000).

### 2.8 Monitoring of PKC pathway activation

Western analysis was performed following the method described in Munro et al. with some modifications (Munro et al., 2007). Mid-log phase cultures were harvested by centrifugation (1500*g*, 5 min, 4 °C) and washed in 1 ml cold lysis buffer (50 mM Tris–HCl pH 7.5, 150 mM NaCl, 0.5% NP40, 2 g ml^−1^ Leupeptin, 2 μg ml^−1^ Pepstatin, 1 mM PMSF, 2 mM Na_3_VO_4_, 50 mM NaF). Cells were collected by centrifugation (800*g*, 5 min, 4 °C) and resuspended in 250 μl cold lysis buffer. Cells were broken using a FastPrep machine in the presence of acid-washed glass beads (4 × 15 s bursts at speed 6.5 with 1 min on ice between bursts). The extracts were clarified by centrifugation (16 000*g*, 5 min, 4 °C). Protein concentration in the cleared lysate was estimated using the method described by Bradford with BSA as a standard (Bradford, 1976).

Proteins were separated by SDS–polyacrylamide gel electrophoresis (SDS–PAGE) using the XCell *SureLock*™ Mini-Cell system (Invitrogen) with NuPAGE^®^Novex Bis–Tris 4–12% pre-cast gels (Invitrogen) in NuPAGE^®^ MOPS-SDS Running Buffer (Invitrogen) containing NuPAGE^®^ Antioxidant (Invitrogen) as per the manufacturer’s instructions. Approximately 15 μg of protein was loaded in each lane. The proteins were transferred to Invitrolon™ PVDF Membranes (Invitrogen) in NuPAGE^®^ Transfer Buffer containing methanol using the XCell II™ Blot Module (Invitrogen) following the manufacturer’s instructions.

Following transfer, the membranes were rinsed in PBS and blocked in PBS-T+10% BSA (PBS, 0.1% Tween 20, 10% (w/v) BSA, 50 mM NaF) for 30 min at RT. The membranes were then incubated overnight at 4 °C in PBS-T+5% BSA (PBS, 0.1% Tween 20, 5% (w/v) BSA, 50 mM NaF) containing a 1:1000 dilution of Phospho-p44/42 MAP kinase (Thr202/Tyr204) antibody (Cell Signaling Technology). The membranes were washed five times for 5 min in PBS−T (PBS, 0.1% Tween 20) and then incubated for 1 h at RT in PBS−T+5% BSA containing a 1:2000 dilution of Anti-rabbit IgG, HRP-linked antibody (Cell Signaling Technology). The membranes were washed three times for 5 min in PBS−T and the signal was detected using LumiGLO™ Reagent and Peroxide (Cell Signaling Technology) as per the manufacturer’s instructions.

### 2.9 Transmission electron microscopy

Mid-exponential phase yeast cells were grown in YPD harvested by centrifugation and the pellets were fixed in 3% glutaraldehyde (v/v) in 0.1 M sodium phosphate buffer pH 7.4. Secondary fixation was performed in 1% osmium tetroxide in dH_2_O and cells were then embedded in TAAB resin before ultrathin silver or gold defracting sections were cut on a Leica UC6 ultramicrotome and stained with uranyl acetate and lead citrate and examined with Philips CM10 transmission microscope (FEI UK Ltd., Cambridge, UK) and the images recorded with a Gatan Bioscan 792 (Gatan UK, Abingdon, UK). Average cell wall thickness was measured manually for at least 20 individual yeast cells.

### 2.10 Analysis of the cell wall β-glucan content

The β-glucan content was determined by hydrolysis of this polymer and quantification of glucose. Yeast cells were grown in YPD medium at 30 °C, broken and hydrolyzed as described (Mora-Montes et al., 2007). Hydrolyzed samples were analyzed by high-performance anion-exchange chromatography with pulsed amperometric detection (HPAEC-PAD) in a carbohydrate analyzer system from Dionex (Surrey, UK), equipped with a ED50 electrochemical detector with gold electrode, a GS50 pump gradient, and a CarboPac PA200 analytical column (3× 250 mm) with a CarboPac PA200 guard column (3× 50 mm). In order to elute the samples, an isocratic gradient of 3.2 mM NaOH with a flux rate of 0.15 ml/min was applied for 20 min. The column was washed with 200 mM NaOH and equilibrated with 3.2 mM NaOH prior to the next analysis.

## 3 Results

### 3.1 Creation of the library

Mutants were constructed using an insertional mutagenesis strategy (Davis et al., 2002) based on the UAU1 marker cassette (Enloe et al., 2000). The UAU1 cassette (*ura*3δ3′-ARG4-*ura*3δ5′) includes a functional *ARG4* gene that can be excised by recombination between flanking repeats of an interrupted *URA3* gene to regenerate a functional *URA3* gene. This cassette, present in the *Tn7*-UAU1 transposon (see Materials and methods), was inserted by *in vitro* transposition into each of the 39 target ORFs encoding putative GpiPs (Fig. 1a). We selected insertions where the UAU1 cassette had inserted near the middle of the ORF. Each of the 39 disruption cassettes was transformed into *C. albicans* strain BWP17 after excision from the vector. Following disruption of the first allele, spontaneous recombination events gave rise to Arg^+^, Ura^+^, His^−^ segregants in which one allele is disrupted by the *ura3-ARG4-ura3* marker and the other one by the recombined *URA3* marker. After PCR diagnostics, at least two mutants per gene were selected for further analysis (Fig. 1a).

One concern with this approach relates to the mitotic recombination or gene conversion step. The selection for homozygosity of the UAU1 insertion may result in homozygosing of part or the entire chromosome carrying the cassette. This may unmask recessive alleles and generate phenotypes not linked to the interruption of the target gene. We believe nevertheless that this bias might be limited since: (i) we tested at least two independent mutants, (ii) in the case of previously studied genes, the phenotypes observed in our strains matched the published ones (*DFG5*, *SSR1*); (iii) when strains obtained by the UAU1 method and the classical Ura-Blaster method were compared (e.g. *PGA31, SSR1*) identical phenotypes were observed.

Our current library, composed of 39 UAU1 insertional mutants plus six mutants provided by different laboratories, represents 39.1% of the 115 putative GpiPs of *C. albicans*. The genes disrupted were not selected using any particular criteria. This library represents the first part of our study of all predicted GpiPs of *C. albicans* and will be followed in time by the characterization of the remaining genes. The construction of each of the 39 mutants, even using the UAU1 method, was time consuming thus we decided to screen this library before completion of all the gene disruptions. With a little less than 40% of the genes deleted we unveiled important roles for some of these GpiPs, giving an overview of the biological importance of these proteins. This is of particular interest since these proteins are predicted to be at the cell surface and more than 60% are of unknown function.

### 3.2 Primary screening on the 45 mutants

This library was initially screened for various biological functions including morphogenesis, ability to respond to diverse stresses, cell wall related phenotypes and susceptibility to antifungal drugs. In this publication, we will mainly focus on the cell wall and how deletion of specific predicted GpiPs affects the structure, composition and resistance to stress of the cell wall. The additional results obtained from the whole screen are supplied in Supplementary data S1.

Thus, in order to evaluate the impact of the disruption of genes encoding putative GpiPs on the structural integrity of the cell wall, mutants were tested for sensitivity to a number of agents that target the cell wall or membrane. These included Calcofluor White (CFW) a fluorescent optical brightener with chitin-binding properties and caspofungin a drug inhibiting the synthesis of β-1,3-glucan, the main component of the cell wall. In addition we measured growth in the presence of SDS a detergent that compromises the integrity of the cell membrane, this tests the accessibility of SDS to the membrane through the cell wall.

**Fig. 2:**
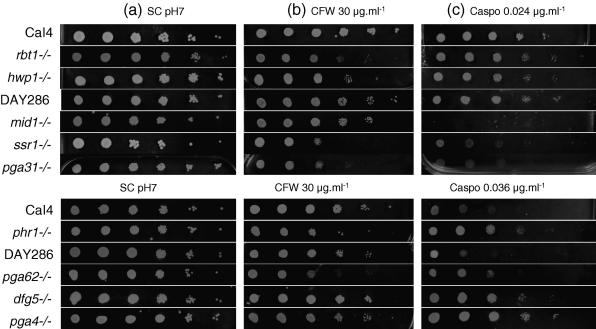
Sensitivity of GpiP mutants to cell wall perturbing agents. The reference and mutant strains were grown on (a) SC medium buffered at pH7, supplemented (b) with 30 μg ml^−1^ Calcofluor White or (c) with 0.024 or 0.036 μg ml^−1^ caspofungin for 2 days at 37 °C. Only mutant strains with a phenotype different from the reference strain are shown. CAI-4 was used as the reference strain for the URA-Blaster mutants and DAY286 for the UAU1 mutants. Ten times serial dilutions of a culture at OD_600_ = 1 were dropped on the plate.

Hypersensitivity to CFW was observed with six mutants *rbt1*−/−, *hwp1*−/−, *pga31*−/−, *pga62*−/−, *phr1*−/− and *ssr1*−/− using serial dilutions on plates containing different concentrations of CFW (Fig. 2). In the presence of SDS, using the same protocol, hypersensitivity was observed with two mutants *pga31*−/− and *hwp1*−/− (data not shown).

In order to evaluate the *in vitro* susceptibility of the GpiP mutants to caspofungin, MICs of caspofungin were determined using the CLSI (former NCCLS) microbroth dilution method M27-A2 (NCCLS, 2002). Several strains had different susceptibilities to caspofungin compared to the reference strain DAY286. The bank of 45 mutants were then screened for altered growth on SC-plates containing caspofungin. Strains *mid1*−/−, *ssr1*−/− and *pga31*−/− were hypersensitive to caspofungin and *pga62*−/−, *dfg5*−/−, *phr1*−/− and *pga4*−/− showed reduced susceptibility (Fig. 2). Out of the 45 mutants tested, 9 mutants were affected by the cell wall perturbing agents including 7 that had altered caspofungin sensitivity in comparison to the parental strain (Table 2).

### 3.3 Analysis of the GpiP mutants with caspofungin-susceptibility phenotypes

The seven mutants with altered caspofungin sensitivity were characterized more precisely by monitoring three distinct features: (i) the overall cell wall architecture using electron microscopic imaging; (ii) activation of the PKC signalling pathway by monitoring the phosphorylation of Mkc1 and (iii) the cell wall chemical composition by quantifying the glucan and chitin content.

### 3.4 Cell wall architecture

**Fig. 3:**
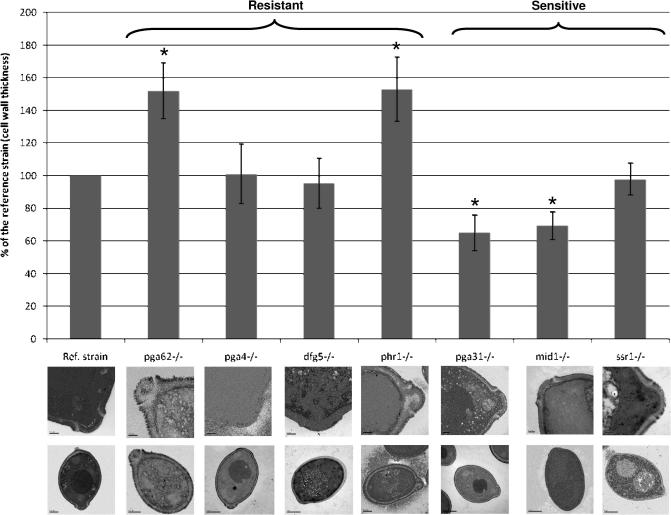
TEM reveals GpiPs mutants have different overall cell wall thicknesses Mid-exponential phase yeast cells grown in YPD were harvested, fixed, sectioned and examined by TEM (wild-type: 0.15 μm). Cell wall thicknesses were measured manually for at least 20 individual yeast cells. The asterisks indicate significantly different thicknesses (*T*-test, *p* < 0.05). Caspofungin-resistant and sensitive strains are clustered on the chart.

Transmission electron microscopy (TEM) was used to look for gross changes in the cell wall structure of the mutants with altered caspofungin sensitivities. Although subtle changes in cell wall architecture may be missed by this approach significant changes in the thickness of the cell wall layer were observed. Strains that were less susceptible to caspofungin had thicker walls (Fig. 3) than the reference strain DAY286, and strains with increased caspofungin susceptibility had thinner walls. In the *phr1*−/− mutant, with a significantly thicker cell wall; the TEM images show an increase of the central low density layer, but not of the peripheral high density layers (mainly composed of mannoproteins) on the outer face of the cell wall. This implies that the chitin/glucan core is affected by the lack of Phr1. This is compatible with the predicted β-1,3-glucanosyl transferase function for Phr1.

### 3.5 Activation of the PKC pathway and caspofungin susceptibility

**Fig. 4:**
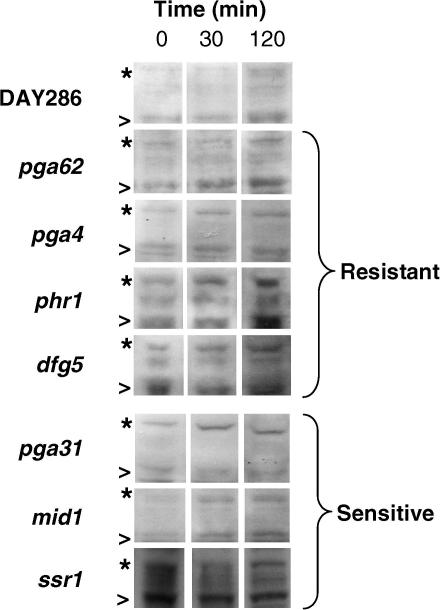
The PKC pathway is activated in GpiP mutants Cell extracts were prepared from cells harvested at different time points (0–120 min), blotted on a membrane and hybridised with anti-phospho Mkc1 and Cek1 antibodies: “∗” and “>” symbolized Mkc1p and Cek1p respectively. Caspofungin-resistant and sensitive strains are indicated.

It has been suggested that *C. albicans* and *S. cerevisiae* respond to caspofungin by activating the protein kinase cell integrity pathway (PKC) (Bruno et al., 2006; Lesage et al., 2004; Liu et al., 2005; Reinoso-Martin et al., 2003; Walker et al., unpublished). In order to investigate this further, we examined by western analysis the phosphorylation status of Mkc1, the MAP kinase of the PKC integrity pathway in the GpiP mutants with altered caspofungin susceptibility (Fig. 4). Mkc1 was phosphorylated in *dfg5*−/−, *phr1*−/− and *pga31*−/− and little or no phosphorylation was observed in *pga4*−/−, *pga62*−/−, *mid1*−/−, *ssr1*−/− and the reference strain DAY286 (Fig. 4). The anti-phospho p42/44 antibody used to monitor Mkc1 phosphorylation serendipitously cross-reacts with the phosphorylated form of Cek1, the MAP kinase of the SVG pathway (Eisman et al., 2006). Cek1 phosphorylation was observed in *phr1*−/− and *pga62*−/− compared to the reference strain. As shown in Fig. 4, phosphorylated Mkc1 was detected in mutants with reduced and elevated susceptibility to caspofungin. This suggests that the differential sensitivities to caspofungin are not due to alterations in the status of the PKC pathway alone.

### 3.6 Measurement of cell wall chitin and glucan content

**Fig. 5:**
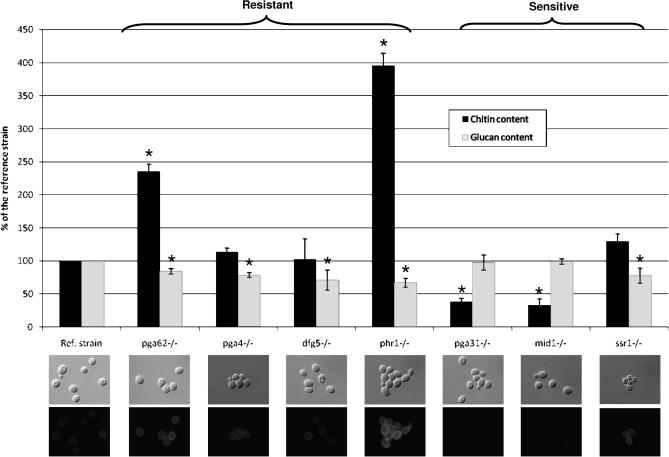
Measurement of cell wall chitin and glucan content Cell wall preparations of three replicates of each strain were subjected to different hydrolysis in order to quantify chitin and glucan levels (see Section 2). The asterisks indicate significant results using the T-test with *p* < 0.05. In addition CFW-stained cells are included as a qualitative measure of cell wall chitin. Caspofungin-resistant and sensitive strains are clustered on the chart.

Altered sensitivities to cell wall perturbing agents indicate modified cell wall properties. The crucial role of the cell wall in fungal viability implies that the cell has developed ways to cope with most alterations of its envelope. It has been shown that one way cells respond to cell wall defects is to modify their cell wall composition (Popolo et al., 2001). We investigated cell wall remodelling in the GpiP mutants by quantifying their cell wall chitin and glucan content. The *phr1*−/− and *pga62*−/− mutants have a significant increase in cell wall chitin content (Fig. 5). In contrast, *mid1*−/− and *pga31*−/− have significantly decreased chitin levels. Whereas, *pga4*−/−, *dfg5*−/− and *ssr1*−/− have wild type chitin levels. In general terms such marked differences were not observed in glucan levels however, *phr1*−/−, *pga62*−/−, *pga4*−/−, *dfg5*−/− and *ssr1*−/− have significantly increased cell wall glucan content, while the mutants *mid1*−/− and *pga31*−/− conserve their wild type glucan levels (Fig. 5).

## 4 Discussion

The ability to disrupt genes rapidly in *C. albicans* is a powerful tool to study gene function. The availability of *C. albicans* genome sequences allows reverse genetics strategies to be employed on a large scale. Equivalent approaches have been carried out to analyse chlamydospore formation, fluconazole resistance and biofilm formation (Bruno and Mitchell, 2005; Nobile et al., 2003; Nobile and Mitchell, 2005; Richard et al., 2005). The approach described here was not focussed on a specific biological process or a definite function, but on a specific cellular component: the cell wall. Using the UAU1 method and the resources of the *Candida* community, we obtained a library of 45 mutants representing almost 40% of the candidate *GPIp* genes identified *in silico* (Richard and Plaine, 2007). This represents the largest library of GpiP mutants and thus is a significant step towards unveiling the roles of all the GpiPs of *C. albicans*. In this study, we considered all the predicted GpiPs but a complete analysis supported by localization and biochemical data will be necessary to ascertain whether these are true GPI-anchored cell wall proteins.

The mutant library was subjected to a series of phenotypic tests that evaluated their ability to grow in a variety of conditions including altered pH, high temperature and oxidative stresses. In total 13 conditions were tested and 15 mutants were identified as showing a phenotype different to the reference strains (see Supplementary Data S1). We observed distinct phenotypes in mutants of only some members of small gene families such as *PGA29/PGA30/PGA31, PGA4/PGA5/PHR1/PHR2* and *HWP1/RBT1/PGA8* suggesting that members of GpiP families have often evolved specific functions. We decided to focus on the mutants affected in cell wall integrity and antifungal sensitivity. Nine mutants tested displayed a phenotype different to the reference strain under at least one condition tested. Moreover, seven of the nine mutant strains exhibited altered caspofungin susceptibility. Further characterization of these mutants revealed significant changes in chitin and glucan levels accompanied by cell wall thickness changes. Activation of the PKC cell integrity signalling pathway was another indication of the important role of GpiPs in maintaining a robust cell wall. Thus, this preliminary survey in *C. albicans* allowed us to identify some novel GpiPs involved in cell wall structure and resistance to cell wall perturbing agents. These putative GpiPs may have a structural role, or may contribute to the biosynthesis or assembly of the major cell wall components or may function in cell wall remodelling.

### 4.1 Calcofluor sensitivity and its relation with cell wall composition

In the majority of cases hypersensitivity to CFW can be correlated with an increase in the cell wall chitin content. Chitin synthesis is activated in order to counteract a weakening of the cell wall as demonstrated in *gpi7*−/− mutants (Richard et al., 2002b). To examine the relationship between CFW sensitivity and elevated chitin content we quantified the chitin content of the mutant strains that were hypersensitive to CFW and examined the level of fluorescence of CFW-stained cells. The *pga62*−/−, *phr1*−/− (Fig. 5) and *rbt1*−/− (L. Walker, N. Gow and C. Munro, unpublished data) mutants had a significant increase in cell wall chitin content. However, *ssr1*−/− (Fig. 5) and *hwp1*−/− (L. Walker, N. Gow and C. Munro, unpublished data) had wild type chitin levels. *pga31*−/− had a significantly decreased chitin content. Cell wall salvage mechanisms have been well-characterised in *S. cerevisiae* (Levin, 2005; Popolo et al., 2001). Defects in cell wall composition and/or structure are sensed by the cell, which consequently trigger a chain of signalling events that lead to the activation of chitin synthesis. For instance, in *S. cerevisiae* the deletion of Gas1 leads to activation of cell wall compensatory mechanisms that result in activation of chitin synthesis and hence CFW hypersensitivity (Popolo et al., 1997; Ram et al., 1998). The Gas family are conserved in other fungi and play critical roles in the remodelling of β-1,3-glucans in the cell wall (Mouyna et al., 2000). In *C. albicans* the Gas-like family is composed of Phr1, Phr2, Pga4/Gas5 and Pga5. In this screen *phr1*−/− was the only mutant of the Gas-like family that was hypersensitive to CFW in the conditions tested. *phr2*−/− and *gas5*−/− showed no sensitivities to SDS or CFW. Phr1 is known to make a key contribution to cell wall integrity acting as a pH dependent transglycosidase and the *phr1*−/− mutant exhibited a clear activation of chitin synthesis (around 4-fold higher chitin than the reference strain) (Fonzi, 1999; Saporito-Irwin et al., 1995). This increase was accompanied by a reduction of the glucan content suggesting a more direct role of the transglycosidase on the cell wall glucan level.

The *pga62*−/− mutant was also hypersensitive to CFW. *PGA62* and its paralog *PGA59*, both encode small GPI-proteins of unknown function. This result suggests that Pga62 is involved in cell wall integrity and that Pga59 cannot compensate for the absence of Pga62 under the conditions tested. Pga59 and Pga62 may be capable of functional redundancy but they may be expressed under completely different conditions thus disabling any functional compensation as is the case for Phr1 and Phr2. The overexpression of Pga59 in a *pga62*−/− mutant background would be an interesting experiment to question the redundancy of function in this small family. The *pga62*−/− mutant in our screen had increased chitin levels and decreased glucan levels, probably as a consequence of cell wall remodelling. Therefore, these results suggest that Pga62 is likely to play a role in cell wall biosynthesis but we cannot define a specific function from this analysis.

As well as sensitivity to elevated temperature (Supplementary data S1), the *ssr1*−/− mutant was hypersensitive to CFW (Garcera et al., 2003). Despite this we measured no change in cell wall chitin levels and only marginally decreased glucan. It has been demonstrated that Ssr1 is associated with cell wall β-glucans and it has been suggested that Ssr1 might participate in the assembly of cell wall components to give the final organization of the cell wall (Garcerá et al., 2005). The cell wall defects of the *ssr1*−/−, reflected in the lower level of glucan, may not be sufficient to trigger the PKC cell wall salvage pathway or activate compensatory chitin synthesis.

Interestingly, in the *pga31*−/− mutant the chitin content was significantly lower than the reference strain despite CFW and SDS hypersensitivity and activation of the PKC pathway (see below), which is unusual knowing the normal course of events during cell wall remodelling. This suggests alternative hypotheses, which are not mutually exclusive, that Pga31 is part of the cell wall salvage pathway or that Pga31 is involved in the regulation or assembly of chitin. In the latter case the decrease of chitin would be a direct consequence of the absence of Pga31, and the hypersensitivity to CFW could be explained by a weakened cell wall in which the low residual chitin content is essential for integrity. The pleiotropic phenotypes of *pga31*−/− (supplementary data S1) suggest an important role in cell wall biosynthesis and integrity. This is supported by the finding that *PGA31* gene expression was induced during protoplast regeneration (Castillo et al., 2006). Pga31 is a member of a three-protein family of unknown function, sharing 59% and 54% amino acid sequence similarity with Pga30 and Pga29, respectively. A mutant lacking *PGA29* is not currently available, but the *pga30*−/− mutant contained in our library, was tested and did not display any of the *pga31*−/− phenotypes. This implies that Pga31 has a unique function that cannot be compensated by its paralogs in the conditions tested.

The *rbt1*−/− (Regulated By Tup1) mutant was hypersensitive to CFW but not to any other cell wall perturbing agent (Fig. 2). Rbt1 has been characterized *in silico* as being cell wall localized (Alberti-Segui et al., 2004) and prior studies indicated that mutants lacking *RBT1* have significantly reduced virulence (Braun et al., 2000). Interestingly, *RBT1* and *HWP1* share high identity over the entire sequence except in the extracellular domain, which in the case of Hwp1, is a substrate for transglutaminases (Staab et al., 1999). Both of these proteins are hypha-induced (Ihmels et al., 2005) and regulated by Tup1 (Braun et al. 2000), and both may have adhesion properties to different substrates. We show here that inactivation of both genes results in CFW hypersensitivity, but *rbt1*−/− was neither hypersensitive to SDS nor affected in hyphal formation contrary to *hwp1*−/− (data not shown). This suggests that the two paralogs perform related, but non-redundant functions. Staab et al. (1999) reported that Hwp1, might mediate the stable attachment of *C. albicans* hyphae to human buccal epithelium cells by acting as a substrate for mammalian transglutaminase. The SDS and CFW hypersensitivity of *hwp1*−/− imply a marked defect in cell wall composition and structure, which may also explain the filamentation defect. In conclusion, there are reasons to suggest that the role of Hwp1 is not only to serve as a substrate for transglutaminase, but also to participate in cell wall architecture and in hyphal differentiation.

### 4.2 Caspofungin susceptibility and its relation to cell wall composition and thickness

Microarray studies reported that caspofungin induces expression of several genes encoding cell wall proteins and cell wall maintenance proteins both in *S. cerevisiae* and *C. albicans* (Bruno et al., 2006; Lesage et al., 2004; Liu et al., 2005; Reinoso-Martin et al., 2003).

Echinocandins target cell wall biosynthesis by inhibiting the production of β-1,3-glucan, which in *S. cerevisiae* and *C. albicans* represents the main structural skeleton of the cell wall to which all other components are cross-linked (Klis et al., 2002). Consequently echinocandins are fungicidal to *C. albicans*. We found no absolute correlation between caspofungin and CFW sensitivity or PKC pathway activation; but we speculate that in some cases at least the strains (phr1−/− and pga62−/−) that were more resistant to caspofungin may have triggered a cell wall salvage mechanism that resulted in elevated chitin levels. Increased chitin synthesis has already been associated with reducing caspofungin susceptibility (Stevens et al., 2006; Walker et al., 2008). The opposite situation is also observed with strains that have a weaker cell wall because they have low chitin content, and thus are more susceptible to caspofungin treatment like pga31−/−, and mid1−/−. However, in other mutants with altered caspofungin sensitivity (dfg5−/−, pga4−/− and ssr1−/−) chitin levels were similar to the parental strain DAY286 (see Fig. 3).

Interestingly, the glucan composition does not give a clear clue to explain the caspofungin susceptibility phenotypes, this may be because the glucan is not the target of caspofungin but the product of the enzyme targeted.

Interestingly, we found the same correlation between caspofungin susceptibility and cell wall thickness and between caspofungin susceptibility and chitin content: *pga31*−/− and *mid1*−/− have a thin cell wall and are sensitive whereas *phr1*−/− and *pga62*−/− have thick cell wall and are more resistant. Consequently, thickness and chitin content are here tightly linked: low chitin content is associated with thin cell wall and vice versa.

We observed that the disruption of *MID1* blocked the growth of the fungus only in presence of caspofungin. Mid1 plays a role in the calcineurin response in *S. cerevisiae* and *C. albicans* (Brand et al., 2007). It is required for the normal response to iron stress in both yeasts (Supplementary data S1). Moreover, in *C. albicans* the growth of calcineurin mutants are affected not only by cell wall disturbing agents but also osmotic stress agents and azole treatment (Sanglard et al., 2003), a phenotype not shared by the *Camid1*−/− mutant. In addition, the very low amount of chitin in the mutant cells indicates that *Camid1*−/− is unable to produce a mature cell wall, suggesting the function of *C. albicans* Mid1 might have diverged from that of *S. cerevisiae*. Our work also suggests an essential and specific role of *CaMid1* in caspofungin tolerance.

In *S. cerevisiae*, the screening of a yeast mutant collection for altered sensitivity to caspofungin identified 39 resistant mutants (Lesage et al., 2004). The genes deleted in these mutants encoded proteins involved in cell wall assembly such as Fks2, Slg1 (a sensor for the cell integrity pathway) and Tus1 (a GDP-GTP exchange factor for Rho1); but no GpiPs were included. In our study, two of the mutants conferring reduced sensitivity to caspofungin belong to components affecting glucan assembly and remodelling: *phr1*−/− and *pga4*−/−. As described above, Phr1 is required for the proper cross-linking of β-1,3- and β-1,6-glucans. These results suggest that *Phr1* and *Pga4* could be additional targets for caspofungin due to their roles in β-1,3-glucan assembly and maturation, which explains the phenotypes observed. The *pga62*−/− and *dfg5*−/− mutants were also less susceptible to caspofungin. *Pga62* and *Dfg5* participate in the maintenance of cell wall integrity but their precise functions remain unknown. *Dfg5* and its paralog *Dcw1* have some homology to bacterial mannosidases and may participate in the translocation of GpiPs from the membrane to the wall. One possibility is that *Pga62* and *Dfg5* mediate effects of the drug or that the effects of cell wall remodelling due to their absence makes the cell more resistant to caspofungin.

## 5 Conclusion

Our results highlight three aspects related to caspofungin sensitivity: (i) chitin levels greatly influence the sensitivity to this drug, high chitin content confers reduced susceptibility while low chitin content confers hypersensitivity; (ii) in the majority of the cases a link can be drawn between thickness of the cell wall, chitin level and sensitivity to caspofungin treatment: thick cell wall have a higher concentration of chitin and confers resistance and *vice versa*; (iii) surprisingly very little correlation can be found between cell wall glucan content or PKC pathway activation and the sensitivity to caspofungin treatment. These observations cannot be generalized for all strains but give new insights in the study of caspofungin resistance.

In terms of gene discovery, this work emphasizes the importance of two new proteins for *C. albicans* cell wall integrity Pga31 and Pga62. Pga31 mutant strain shows pleiotropic phenotypes with large modifications in cell wall composition and caspofungin sensitivity, suggesting a function in the early steps of cell wall biosynthesis or in cell wall salvage pathways. The absence of Pga62 strongly alters the cell structure and composition in the opposite way to Pga31 and consequently renders the strain more resistant to caspofungin. The less pronounced phenotypes suggest a function in the late stage of cell wall assembly. This library allowed the identification of three genes that when deleted lead to increased caspofungin sensitivity *SSR1*, *MID1* and *PGA31*. We also identified four genes that when deleted lead to decreased caspofungin sensitivity: *DFG5, PHR1, PGA4* and *PGA62*. In the literature, no other examples of deletion mutants resistant to caspofungin have been found in *C. albicans* apart from strains with point mutations in the *FKS1* target gene (Park et al., 2005). This work gives new leads to study cell wall assembly mechanisms and caspofungin resistance emergence.

## Figures and Tables

**Table 1:** Mutant strains used in this study

Gene name	Orf19 number	Similarity group	Function	Source
CHT1	7517	1	Chitinase	This work
CHT2	3895	1	Chitinase	This work
PGA37	3923	2	Putative GPI-anchored protein of unknown function	This work
PGA57	4689	2	Putative GPI-anchored protein of unknown function	This work
SAP9	6928	3	aspartyl proteinase, yapsin	This work
SAP10	3839	3	aspartyl proteinase, yapsin	Albrecht et al. (2006)
PGA2/SOD4	2062	4	Copper- and zinc-containing superoxide dismutase	This work
PGA3/SOD5	2060	4	Copper- and zinc-containing superoxide dismutase	Fradin et al. (2005)
PGA9/SOD6	2108	4	Copper- and zinc-containing superoxide dismutase	This work
HWP1	1321	5	Hyphal cell wall protein	Staab et al. (1999)
RBT1	1327	5	Putative cell wall protein with similarity to Hwp1	Braun et al. (2000)
PGA8	3380	5	Putative GPI-anchored protein of unknown function	This work
PGA30	5303	6	Putative GPI-anchored protein of unknown function	This work
PGA31	5302	6	Putative GPI-anchored protein of unknown function	This work and Santendreu’s laboratory (unpublished)
PGA32	6784	6	Putative GPI-anchored protein of unknown function	This work
CSA1	7114	7	Surface antigen on elongating hyphae and buds	This work and Lamarre et al. (2000)
PGA7	5635	7	Putative GPI-anchored protein of unknown function	This work
PGA10	5674	7	Putative GPI-anchored protein of unknown function	This work
RBT5	5636	7	GPI-anchored cell wall protein	This work and Braun et al. (2000)
PGA4	4035	8	Putative GPI-anchored protein of unknown function	This work
PGA5	3693	8	Putative GPI-anchored protein of unknown function	This work
PHR1	3829	8	Glycosidase of hyphal cell surface	Saporito-Irwin et al. (1995)
PHR2	6081	8	Glycosidase; role in cell wall structure	Fonzi et al. (1999)
DFG5	2075	NS	N-linked mannoprotein of cell wall and membrane	This work
ECM331	4255	NS	Putative GPI-anchored protein of unknown function	This work
HYR1	4975	NS	Nonessential, GPI anchored, predicted cell wall protein	This work and Bailey et al. (1996)
MID1	3212	NS	Putative component of the high affinity calcium uptake system	This work
PGA6	4765	NS	Putative GPI-anchored protein of unknown function	This work
PGA17	893	NS	Putative GPI-anchored protein of unknown function	This work
PGA23	3740	NS	Putative GPI-anchored protein of unknown function	This work
PGA24	3816	NS	Putative GPI-anchored protein of unknown function	This work
PGA27	2044	NS	Putative GPI-anchored protein of unknown function	This work
PGA33	876	NS	Putative GPI-anchored protein of unknown function	This work
PGA36	5760	NS	Putative GPI-anchored protein of unknown function	This work
PGA39	6302	NS	Putative GPI-anchored protein of unknown function	This work
PGA40	1616	NS	Putative GPI-anchored protein of unknown function	This work
PGA42	2907	NS	Putative GPI-anchored protein of unknown function	This work
PGA43	2910	NS	Putative GPI-anchored protein of unknown function	This work
PGA45	2451	NS	Putative GPI-anchored protein of unknown function	This work
PGA46	3638	NS	Putative GPI-anchored protein of unknown function	This work
PGA50	1824	NS	Putative GPI-anchored protein of unknown function	This work
PGA55	207	NS	Putative GPI-anchored protein of unknown function	This work
PGA62	2765	NS	Putative GPI-anchored protein of unknown function	This work
SPR1	2237	NS	Similar to ScSpr1, a sporulation-specific exo-1,3-beta-glucanase	This work
SSR1	7030	NS	Beta-glucan associated cell-wall protein	This work and Garcera et al. (2003)

Similarity group: genes are classified in the table into 8 families according to their amino acid sequence similarity; NS means no similarity within the groups of genes.

**Table 2:** Categories of mutants obtained from Calcofluor white and caspofungin screen

Category	Description of mutant	Interrupted gene
I	Hypersensitive to CFW	*RBT1, HWP1*
II	Hypersensitive to caspofungin	*MID1*
III	Hypersensitive to CFW and caspofungin	*PGA31, SSR1*
IV	Hypersensitive to CFW and resistant to caspofungin	*PGA62, PHR1*
V	Only resistant to caspofungin	*DFG5, PGA4*
