# Supplementary material for: Functional analysis of Candida albicans GPI-anchored proteins: Roles in cell wall integrity and caspofungin sensitivity
Source: Fungal Genet Biol. 2008 Oct;45(10-2):1404–14. doi: 10.1016/j.fgb.2008.08.003 (PMC2649418; doi:10.1016/j.fgb.2008.08.003)
Supplement: Supplementary Data [file mmc1.doc]

**Supplementary data S1**: Complete results of the GpiPs mutant library phenotypic analysis

| Interrupted **gene** | Tested on: |  |  |  |  |  |  |  |  |  |  |  |  | Source |
| --- | --- | --- | --- | --- | --- | --- | --- | --- | --- | --- | --- | --- | --- | --- |
|  | **NaCl** | **H_2_O_2_** | **Fe3^+^** | **CH_3-_COOH** | **Cd** | **43.5°C** | **pH3** | **pH10** | **SDS** | CFW | **Serum** | **Spider** | Caspo |  |
| **Orf19.3893**  ***SAP10*** |  |  |  | GD |  |  |  |  |  |  |  |  |  | (Albrec*ht et a*l., 2006) |
| **Orf19.6928**  ***SAP9*** |  |  |  |  | GD |  |  |  |  |  |  |  |  | This work |
| **Orf19.2060**  ***SOD5*** | GD |  |  |  |  |  |  |  |  |  |  |  |  | (Frad*in et a*l., 2005) |
| **Orf19.2108**  ***SOD6*** |  |  |  |  |  | GD |  |  |  |  |  | Fil- |  | This work |
| **Orf19.1321**  ***HWP1*** |  |  |  |  |  |  |  |  | GD | GD | Fil+/- | Fil- |  | (Sta*ab et a*l., 1999) |
| **Orf19.3829**  ***PHR1*** |  |  |  |  |  |  |  | GD |  | GD | Fil- | Fil- | Tolerant | (Saporito-Irw*in et a*l., 1995) |
| **Orf19.6081**  ***PHR2*** |  |  |  |  |  |  | GD |  |  |  |  |  |  | (Fonzi, 1999) |
| **Orf19.2075**  ***DFG5*** |  |  |  |  |  |  |  |  |  |  | Fil+/- | Fil+/- | Tolerant | This work |
| **Orf19.5302**  ***PGA31*** | GD |  | GD |  |  |  |  |  | GD | GD |  |  | GD | Santendreu |
| **Orf19.5302**  ***PGA31*** | GD |  | GD |  |  |  |  |  | GD | GD |  |  | GD | This work |
| **Orf19.2765**  ***PGA62*** |  |  |  |  |  |  |  |  |  | GD |  |  | Tolerant | This work |
| **Orf19.1327**  ***RBT1*** |  |  |  |  |  |  |  |  |  | GD |  |  |  | (Bra*un et a*l., 2000) |
| **Orf19.4035**  ***GAS1/PGA4*** |  |  |  |  |  |  |  |  |  |  |  |  | Tolerant | This work |
| **Orf19.3212**  ***MID1*** |  |  | GD |  |  |  |  |  |  |  |  |  | GD | This work |
| **Orf.7030**  ***SSR1*** |  |  |  |  |  | GD |  |  |  | GD |  |  | GD | (Garce*ra et a*l., 2003) |
| **Orf.7030**  ***SSR1*** |  |  |  |  |  | GD |  |  |  | GD |  |  | GD | This work |
| **Orf.7114**  ***CSA1*** |  | GD |  |  |  |  |  |  |  |  |  |  |  | (Lamar*re et a*l., 2000) |
| **Orf.7114**  ***CSA1*** |  | GD |  |  |  |  |  |  |  |  |  |  |  | This work |

GD: growth defect

**Material and methods**

The phenotypes of the 45 GpiP mutants were monitored using drop tests. All media were supplemented with histidine. In addition filamentation inducing media were supplemented with 25 µg mL^-1^ uridine. Strains were grown overnight in YPD at 30 °C and diluted with H_2_O to an OD_600_ of 1. Then, 5 µl of serial 1/10 dilutions was spotted onto the following solid media: YPD supplemented with either 1.25 M NaCl, 0.025% SDS or 30 µg Calcofluor White mL^-1^ (Sigma-ALDRICH, Steinheim Germany), SC (buffered at pH 7.0 with 100mM Hepes) with either 250 µM Cadmium, 20 mM Iron (FeCl_2_), 7.5 mM H_2_O_2_ and SC buffered at either pH 3.0 (50 mM acid citric and sodium citrate) or pH 10 (NaOH + 50 mM Glycine) and pH 3.0 with 25 mM acetic acid. Plates were incubated for two days at 37 °C, except for one SC pH 7.0 plate that was incubated at 43.5 °C. Filamentation on solid 2% agar medium was tested on either Spider medium (1% nutrient broth, 1% mannitol, 0.2% K_2_HPO_4_) or serum medium (5% horse serum; Sigma-ALDRICH, Steinheim, Germany). Plates were monitored over five days for hypha formation. Amphotericin B, Fluconazole or Chlamydospore assays gave wild type phenotypes for all the strains tested.

**References**

Albrecht, A., et al., 2006. Glycosylphosphatidylinositol-anchored Proteases of *Candida albicans* Target Proteins Necessary for Both Cellular Processes and Host-Pathogen Interactions. J Biol Chem. 281**,** 688-694.

Braun, B. R., et al., 2000. Identification and characterization of *TUP1*-regulated genes in *Candida albicans*. Genetics. 156**,** 31-44.

Fonzi, W. A., 1999. *PHR1* and *PHR2* of *Candida albicans* encode putative glycosidases required for proper cross-linking of beta-1,3- and beta-1,6-glucans. J Bacteriol. 181**,** 7070-9.

Fradin, C., et al., 2005. Granulocytes govern the transcriptional response, morphology and proliferation of *Candida albicans* in human blood. Mol Microbiol. 56**,** 397-415.

Garcera, A., et al., 2003. Identification and study of a *Candida albicans* protein homologous to Saccharomyces cerevisiae Ssr1p, an internal cell-wall protein. Microbiology. 149**,** 2137-45.

Lamarre, C., et al., 2000. Expression cloning of the *Candida albicans* *CSA1* gene encoding a mycelial surface antigen by sorting of *Saccharomyces cerevisiae* transformants with monoclonal antibody-coated magnetic beads. Mol Microbiol. 35**,** 444-53.

Saporito-Irwin, S. M., et al., 1995. *PHR1*, a pH-regulated gene of *Candida albicans*, is required for morphogenesis. Mol Cell Biol. 15**,** 601-13.

Staab, J. F., et al., 1999. Adhesive and mammalian transglutaminase substrate properties of *Candida albicans* Hwp1. Science. 283**,** 1535-8.
